# Rehabilitation of severe dentofacial deformity after early radiotherapy of retinoblastoma: a case report

**DOI:** 10.1186/s13256-023-03761-z

**Published:** 2023-03-09

**Authors:** So-Hyun Kim, Tae-Hyun Choi, You-Sun Lee, Young-Kyun Kim, Deuk-Won Jo, Baek-Kyu Kim, He-Li Choi, Nam-Ki Lee

**Affiliations:** 1 Department of Orthodontics, Section of Dentistry, Seoul National University Bundang Hospital, Seongnam, Republic of Korea; 2 Department of Orthodontics, Korea University Anam Hospital, Seoul, Republic of Korea; 3 Department of Oral and Maxillofacial Surgery, Section of Dentistry, Seoul National University Bundang Hospital, Seongnam, Republic of Korea; 4 Department of Prosthodontics, Section of Dentistry, Seoul National University Bundang Hospital, Seongnam, Republic of Korea; 5 Department of Plastic and Reconstructive Surgery, Seoul National University Bundang Hospital, Seoul National University College of Medicine, Seongnam, Republic of Korea

**Keywords:** Retinoblastoma, Dentofacial deformities, Interdisciplinary treatment, Oral rehabilitation, Facial esthetics, Two-jaw surgery

## Abstract

**Background:**

Retinoblastoma is an intraocular cancer of infancy and childhood, which has been treated with radiation therapy and chemotherapy. Radiation on growing patients can cause deterioration in maxillofacial growth and development that leads to severe skeletal discrepancies between the maxilla and mandible, and dental problems such as crossbite, openbite, and hypodontia.

**Case presentation:**

We present the case of a 19-year-old Korean man with chewing disability and dentofacial deformities. He had undergone enucleation of the right eye and radiation therapy of the left eye due to retinoblastoma 100 days after birth. Subsequently, he received cancer therapy for the secondary nasopharyngeal cancer at the age of 11 years. He was diagnosed with severe skeletal deformity including sagittal, transverse, and vertical growth deficiency of the maxilla and midface, and with class III malocclusion, severe anterior and posterior crossbite, posterior openbite, multiple missing upper incisors, right premolars, and second molars, and impaction of the lower right second molars. To restore impaired functions and esthetics of the jaw and dentition, the orthodontic treatment combined with two jaw surgery was performed. At the end of surgical orthodontics, dental implants were placed for prosthetic treatment of missing teeth. Additional plastic surgery for zygoma elevation was done with calvarial bone graft followed by fat graft. Facial esthetics and occlusal functions of patient were favorably enhanced with the improvement of skeletal discrepancy and the rehabilitation of maxillary dentition by prosthetic work. At the 2-year follow-up, the skeletal and dental relationships and implant prosthetics were well maintained.

**Conclusion:**

In an adult patient with dentofacial deformities caused by early cancer therapy in the head and neck area, interdisciplinary interventions including additional plastic surgery of zygoma depression and prosthetic work of missing teeth as well as surgical–orthodontic treatment could establish favorable facial esthetics and oral rehabilitation.

## Background

Retinoblastoma is the most common intraocular neoplasm in the very young and accounts for 3% of all childhood malignancies [1]. Chemotherapy and radiation therapy (RT) as well as enucleation have been used for treating this eye cancer. However, radiation in pediatric patients with head and neck tumors can damage blood vessels within the bone marrow and impede the growth and development of craniofacial structures [2]. Additionally, dental abnormalities, such as microdontia and hypodontia, are known to develop in patients who have received RT and chemotherapy during childhood [3].

These functional and aesthetic impairments can cause physical, physiological, and social problems in patients, such as craniofacial deformities [4]. To resolve these problems, clinicians might consider orthodontic and prosthodontic treatment with surgical intervention depending on the patient’s status.

A few studies reported a rare case of 11-/12-year-olds with osteosarcoma of the nasopharynx who had been treated with irradiation and chemotherapy for unilateral non-hereditary retinoblasma [5, 6]. This report introduces the case of an adult patient with severe dentofacial deformities, who had undergone enucleation and RT for early bilateral retinoblastoma and cancer therapy for nasopharyngeal cancer at 11 years old, and describes the interdisciplinary treatment by performed to correct his skeletodental anomalies.

## Case presentation

A 19-year-old Korean man presented to department of orthodontics, Seoul National University Bundang Hospital with chief complaints of chewing disability, midfacial depression, and mandibular prognathism. He had no psychosocial history.

As previous medical history, he was diagnosed as bilateral retinoblastoma at 1 month of birth, and had undergone enucleation of the right eye and external beam radiotherapy of the left eye 100 days after birth. He had no hereditary or family history of retinoblastoma. At regular follow-up over 9 years, there was no evidence of recurrence. However, he subsequently received cancer therapy for the secondary nasopharyngeal cancer, with no involvement in other parts, at the age of 11 years in other hospital.

### Diagnosis

**Fig. 1:**
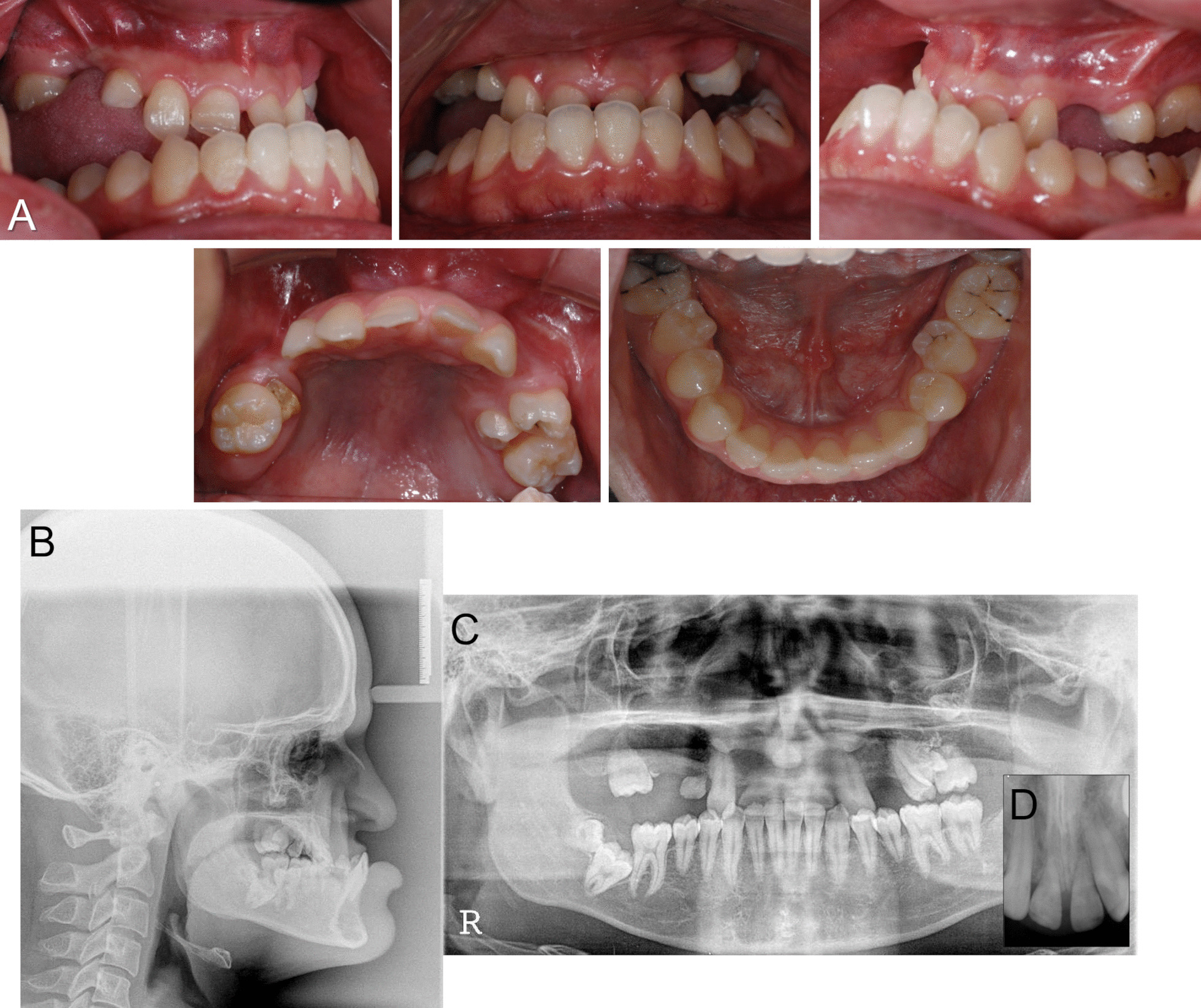
Pretreatment records of the patient. **A**. Intraoral photograph; **B**. Lateral cephalogram; **C**. Panoramic radiograph; **D**. Periapical radiograph

**Fig. 2:**
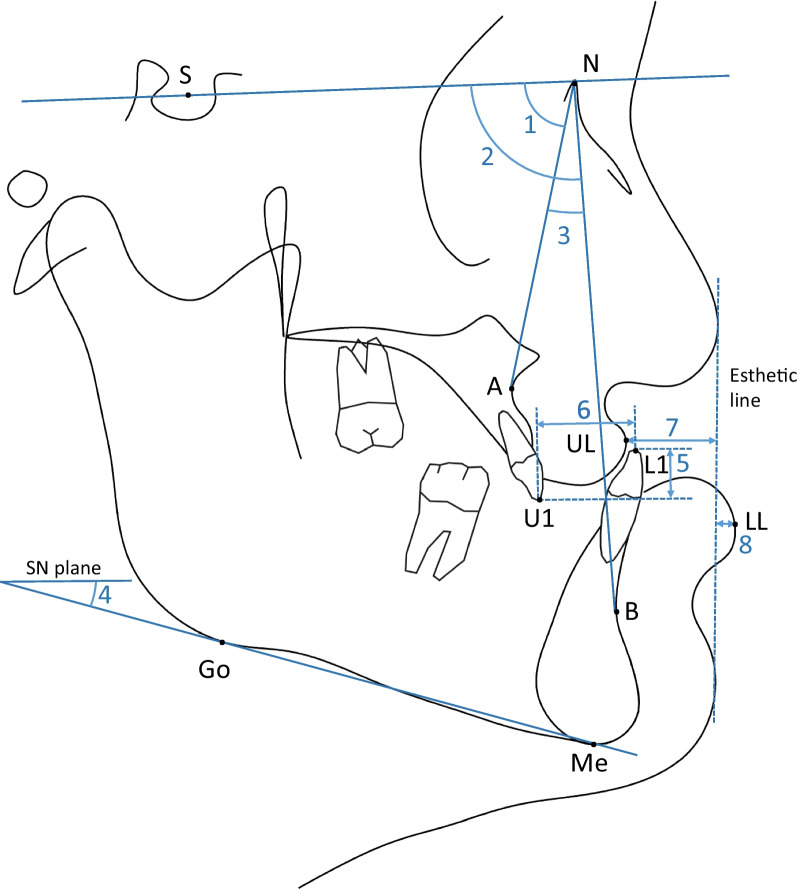
Cephalometric analysis for skeletal, dental, and soft tissue measurements. (1) *SNA* sella–nasion–A point angle; (2) *SNB* sella–nasion–B point angle; (3) *ANB* A point–nasion–B point angle; (4) *SN-GoMe* angle between sella–nasion line and gonion–menton line (Go-Me, mandibular plane); (5) *Overbite* vertical distance between incisal tips of upper central incisor (U1) and lower central incisor (L1); (6) *Overjet* horizontal distance between incisal tips of upper and lower central incisor; (7) *UL to esthetic line* distance between upper lip (UL) to esthetic line (nose tip to soft tissue pogonion); (8) *LL to esthetic line* distance between lower lip (LL) to esthetic line

Extraoral examination and cephalometric radiograph analysis demonstrated skeletal class III with significant sagittal, transverse, and vertical growth deficiency of the maxilla and midface [Sella–nasion–A point angle (SNA), 75.8°; A point–nasion–B point angle (ANB), −16.2°; Table 1 and Figs. 1 and 2]. Intraoral examination revealed angle class III malocclusion, severe anterior and posterior crossbite, and posterior openbite. Panoramic radiograph showed multiple missing upper permanent teeth (incisors, right premolars, and second molars), impaction of the lower right second and third molars, prolonged retention of the primary teeth (central incisors and right canine), and root rest of the upper right primary molar. There was also vertical and horizontal alveolar bone atrophy in the edentulous area of the maxilla (Fig. 1).

**Table 1:** Comparison of cephalometric measurements (pretreatment, posttreatment, and 2-year follow-up)

Variable	Measurement	Normal	Pretreatment	Posttreatment	2-Year follow-up
Skeletal	SNA (°)	82.4	75.75	81.10	80.82
	SNB (°)	80.4	91.9	81.49	81.35
	ANB (°)	2.0	−16.15	−0.4	−0.53
	SN-GoMe (°)	32.0	17.64	27.85	27.40
Dental	Overbite (mm)	2.3	10.73	2.04	1.78
	Overjet (mm)	3.2	−15.87	3.32	3.19
Soft tissue	UL to esthetic line (mm)	1.0	−13.95	−0.93	−1.16
	LL to esthetic line (mm)	1.0	3.29	−1.00	−2.32

For definition of measurements, see Fig. 2

*SNA*, sella-nasion-A point angle; *SNB*, sella-nasion-B point angle; *ANB*, A point-nasion-B point angle; SN-GoMe, angle between sella-nasion line and gonion-menton line; UL to esthetic line, distance between upper lip to nose tip-soft tissue pogonion line; LL to esthetic line, distance between lower lip to esthetic line

### Treatment objectives and plan

The treatment objective was to restore impaired functions and improve esthetics of the jaw and dentition by orthodontic treatment combined with orthognathic surgery, prosthetic treatment, and additional plastic surgery (Table 2).

**Table 2:** Problem list, treatment objectives, and treatment plan

Problem list	Treatment objective	Treatment plan
Maxillary deficiency		
Anteroposterior		(1) Orthodontic treatment
Vertical	To correct skeletal disharmony	(2) Orthognathic surgery (two-jaw surgery, no surgical widening)
Transverse		
Missing teeth	To regain space	(1) Orthodontic treatment
	To restore occlusion	(2) Implants and prostheses
Posterior openbite	To restore vertical relation of occlusion	Surgery (posterior maxillary segmental osteotomy) Prostheses
Impacted teeth and root rest		Extraction
Depression of zygomatic arch and right enophthalmos	To restore facial harmony	Plastic surgery (zygoma elevation and calvarial bone and fat graft)

### Treatment progress and result

**Fig. 3:**
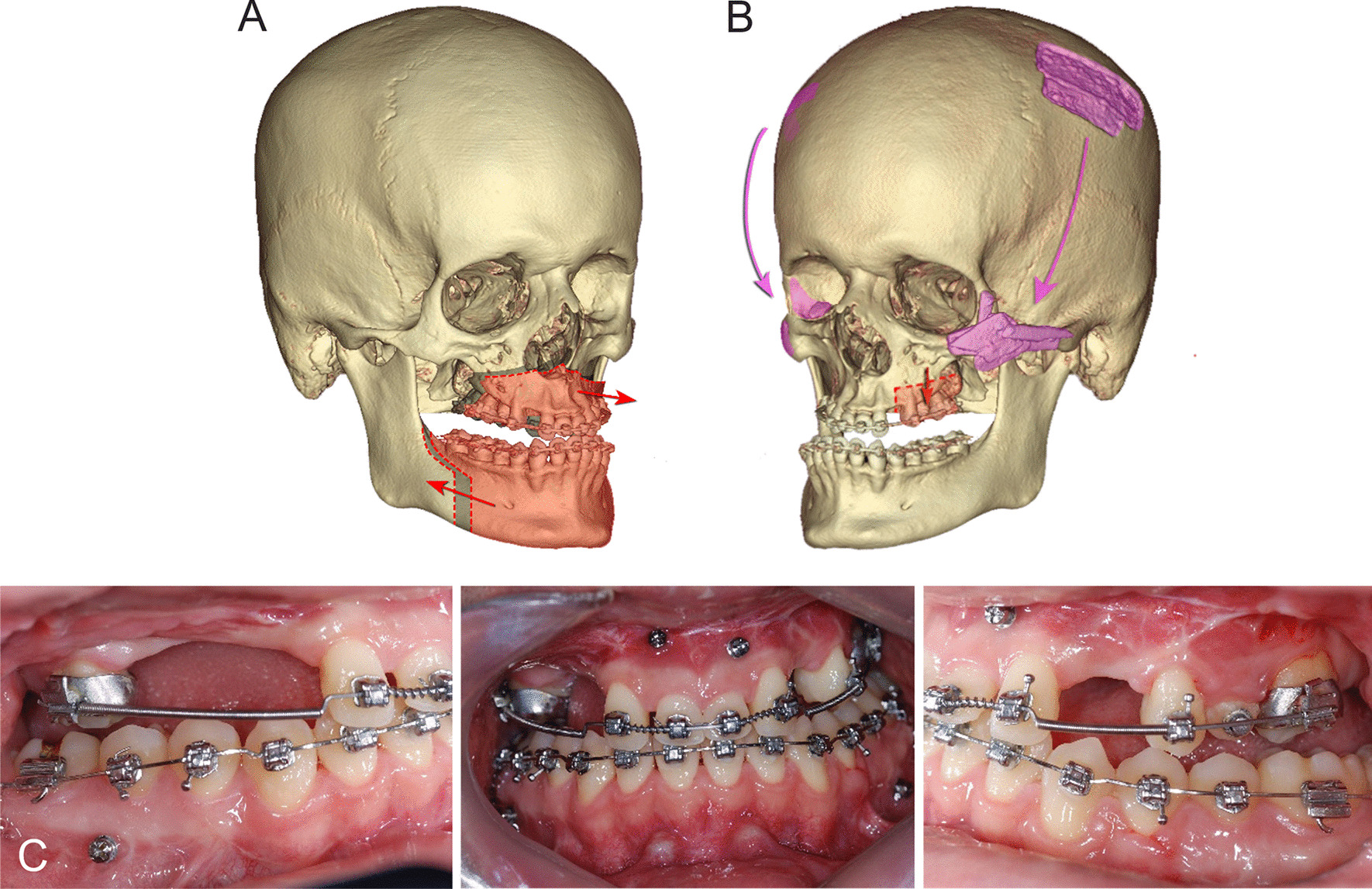
Illustration of surgery and postsurgical orthodontic treatment. **A** Surgical plan for two-jaw surgery; **B** surgical plan for posterior maxillary segmental osteotomy (PMSO) and correction of depressed zygoma and enophthalmos; **C** intraoral photograph after two-jaw surgery and PMSO

**Fig. 4:**
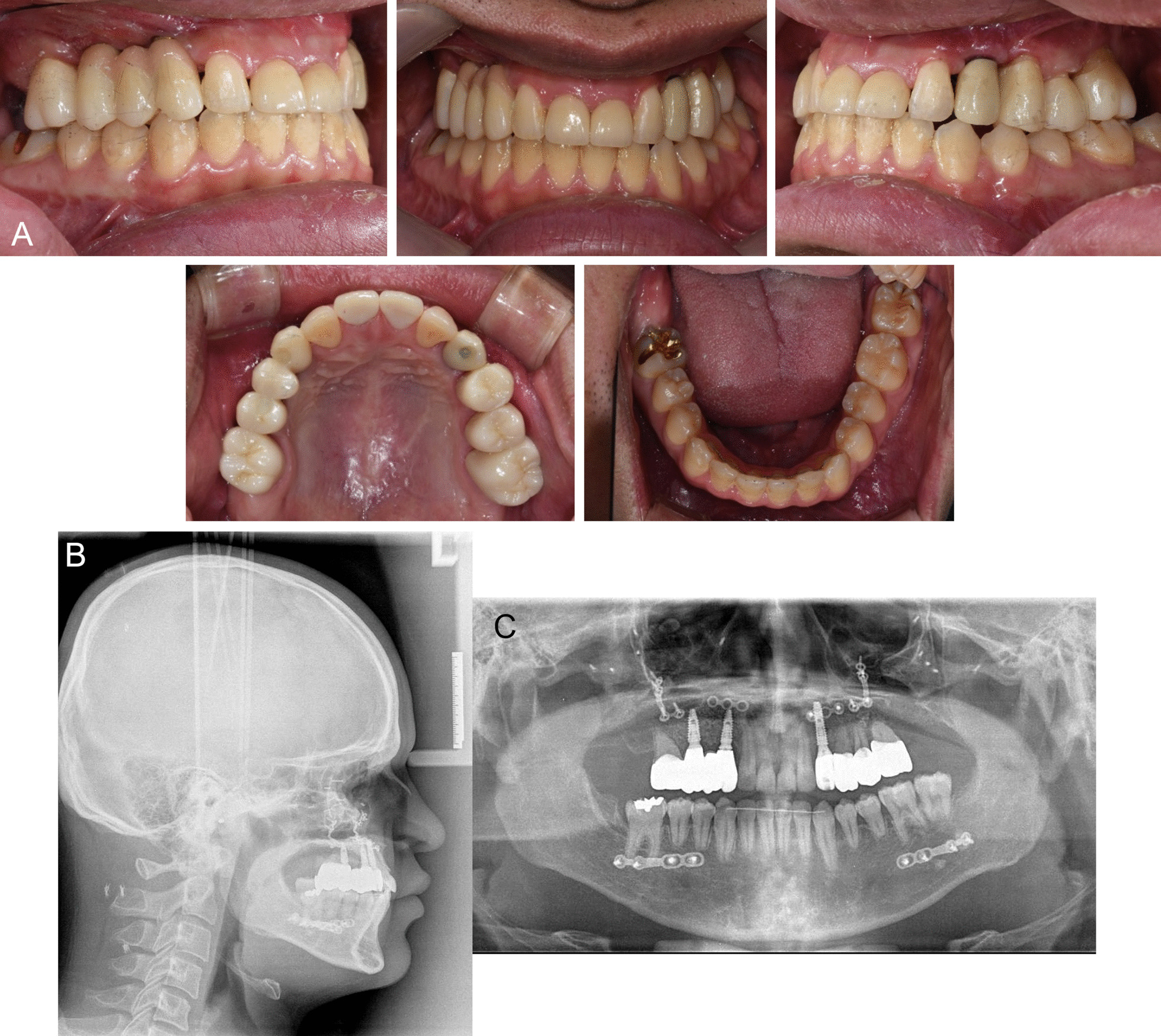
Posttreatment records of the patient. **A**. Intraoral photograph; **B**. Lateral cephalogram; **C**. Panoramic radiograph

**Fig. 5:**
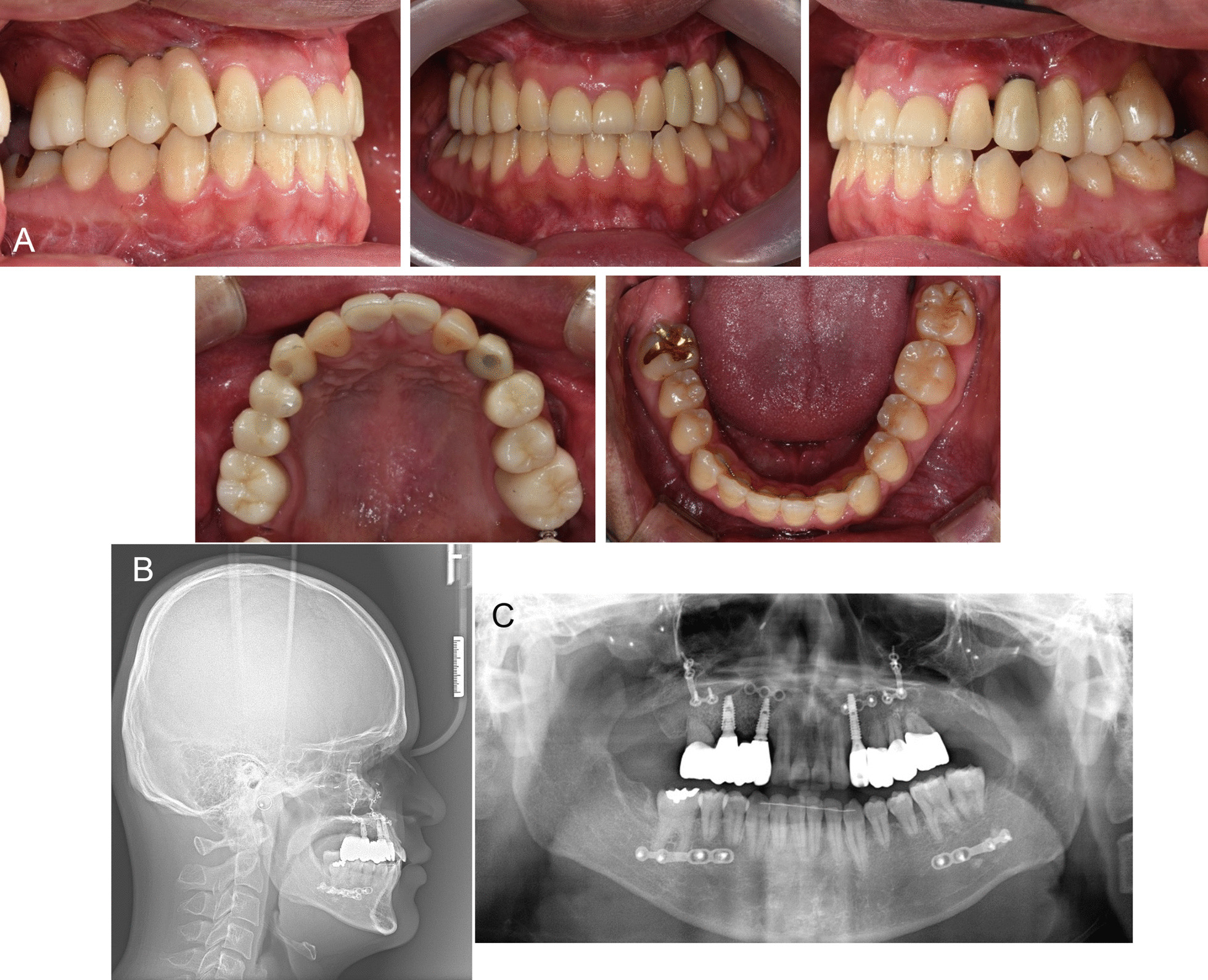
Two-year follow-up records of the patient. **A**. Intraoral photograph; **B**. Lateral cephalogram; **C**. Panoramic radiograph.

The impacted lower right second and third molars were extracted.
Initiation: presurgical orthodontic treatment was performed for dental decompensation and tooth alignment. The space for dental implants between the right maxillary canine and second premolar was regained by extraction of the right primary canine and primary molar root rest.
At 7 months, to correct severe skeletal discrepancies between the maxilla and mandible, orthognathic surgery was performed with maxillary advancement by Le Fort I osteotomy (right, 6 mm; left, 4 mm) and mandibular setback by sagittal split ramus osteotomy (right, 12 mm; left, 15 mm) (Fig. 3A). Intermaxillary fixation was performed with elastics and miniscrew implants for 2 weeks.
At 8 months (postoperative 1 month), postsurgical orthodontic treatment was initiated to stabilize the occlusion and correct the mandibular occlusal plane. Despite skeletal and dental improvement in the sagittal and vertical dimensions, posterior openbite remained because of the insufficient alveolar bone height in the maxillary posterior area. At 40 months (postoperative 33 months), bilateral posterior maxillary segmental osteotomy (PMSO) with right posterior horizontal ridge augmentation using NovaBone (NovaBone Products, Alachua, FL) was additionally performed to increase the vertical height of the posterior alveolar ridge (Fig. 3B, C).
At 43 months (postoperative 36 months), additional reconstructive surgery was performed by a plastic surgeon according to the patient’s demand. Depressed zygomatic arch and right enophthalmos were improved by bilateral zygoma elevation with calvarial bone graft followed by fat graft from abdomen (Fig. 3B). Examination of visual acuity test and exophthalmometry before plastic surgery were also carried out.
At 60 months, orthodontic appliances were removed at the end of the surgical–orthodontic treatment. For prosthetic works, from 57 to 70 months, zirconia crowns for the upper right/left first molars and left premolars were placed by a prosthodontist to resolve the remaining posterior openbite and to complete the occlusion. Three implants (CMI, Neobiotech, Seoul, South Korea) were placed with bone augmentation in the maxillary left/ right canines (width, 3.5 mm; length, 8.5 mm/10 mm), and right second premolar areas (width, 4.0 mm; length, 10 mm) to replace the multiple missing teeth. A zirconia fixed partial denture was placed between the right canine and premolar implants, and a zirconia crown was placed at the left canine implant. Finally, zirconia crowns on the primary central incisors were set for missing maxillary central incisors.
Facial esthetics and functions were favorably enhanced with the improvement of occlusion and skeletal discrepancy. Skeletally, maxillary deficiency and mandibular prognathism were resolved (SNA, 81.1°; ANB, −0.4°; Table 1). Dentally, appropriate overjet (3.3 mm) and overbite (2 mm) were achieved. The occlusal relationship was improved following the rehabilitation of maxillary dentition by prosthetic work (Fig. 4).
(8) At the 2-year follow-up, the skeletal and dental relationships and implant prosthetics were well maintained (Table 1 and Fig. 5).

## Discussion and conclusions

Retinoblastoma is rare intraocular cancer in children. It is a commonly considered to be nonhereditary if a single eye is involved, and about one-third of cases are bilateral [7]. Patients with hereditary retinoblastoma who received RT are reported to have a 35% risk of second cancers, which are more likely to involve the pineal gland, bone (osteosarcoma), soft tissue sarcomas, and melanoma [8]. This report is unique in that the patient was a bilateral but nonheritable case and showed secondary involvement to nasopharynx.

RT in the head and neck tumors cause deterioration of maxillofacial growth and development in growing patients and lead to various problems, such as mastication dysfunction, unesthetic appearance, and loss of self-confidence as well as vision disorders. Martin *et al.* previously reported an 18-year-old patient treated with surgically assisted maxillary expansion by transpalatal distractor followed by maxillary advancement with Lefort I osteotomy implant-supported fixed bridge, who had dentofacial anomaly by previous external RT of bilateral retinoblastoma [9]. Our case showed facial reconstruction and full mouth rehabilitation in an adult patient with the history of enucleation and RT for early bilateral retinoblastoma and cancer therapy for secondary nasopharyngeal cancer.

The most important consideration in treatment planning for this case was how to restore deficiency of the maxilla and underdeveloped alveolar bone height caused by growth disturbance in the midface and maxillary dentition after RT to the left eye and nasopharyngeal area.

The maxilla showed three-dimensional underdevelopment: transverse, anteroposterior, and vertical dimensions. Considering the transverse and vertical deficiencies in the maxilla, maxillary expansion and downward movement were likely necessary. Actual transverse difference between the normal (25.2 mm) and the patient’s maxilla–mandibular differential index (30.2 mm) was approximately 5 mm in the posteroanterior cephalogram [10]. This discrepancy was considered to be clinically acceptable after the sagittal correction of the jaws. Moreover, downward movement or surgical widening of the maxilla has less postsurgical stability [11]. Therefore, anteroposterior skeletal discrepancy and vertical facial dimension were corrected with bimaxillary surgery, including maxillary advancement with mandibular setback.

Presurgical orthodontic treatment was performed in advance to decompensate malocclusion for favorable orthognathic surgery and to regain space for multiple prostheses.

Postoperatively, despite sagittal improvement in the jaws and increased facial height, openbite of the posterior teeth was predicted. The interocclusal gap in the posterior area was approximately 5 mm. Considering the biological limitations in orthodontic posterior extrusion and prosthetic treatment from relatively deficient alveolar ridge height, at least 3 mm of bone augmentation was needed, so PMSO with block bone graft from the ramus was performed before prosthetic rehabilitation of the posterior teeth (Fig. 3C). PMSO with interpositional bone graft is applied to areas requiring ≥ 4 mm bone height augmentation and indicated in cases with posterior maxillary alveolar hypoplasia, posterior openbite, and transverse deficiency [12]. Alveolar bone distraction is another alternative for moderate bone augmentation; however, it has innate disadvantages, such as discomfort and high cost; moreover, the affected area is bilateral.

In addition, this report describes an adult case of severe maxillary retrusion and provides insight into treatment for a growing patient with a similar medical history. Disturbance of craniofacial bone growth occurs in 66–100% of children with head and neck cancer, especially orodental development, resulting in maxillary retrusion, agenesis, and delayed eruption [2, 13].

If this patient had been referred for dentofacial orthopedic treatment during the growth period after medical therapy of secondary nasopharyngeal cancer, maxillary protraction using temporary anchorage device, such as miniplates, would have helped to alleviate the severe maxillary deficiency [14]. On *et al.* reported a 4.8 mm maxillary advancement in growing patients with cleft lip and palate using a facemask with a miniplate [15].

In this case, appropriate diagnosis and timely interdisciplinary approaches contributed to patient’s satisfaction with treatment outcomes. Skeletal and dental relationships were well maintained at 2 years of follow-up, and long-term stability needs to be evaluated.

In conclusion, in an adult patient with dentofacial deformities caused by early cancer therapy in the head and neck area, interdisciplinary interventions including additional plastic surgery of zygoma depression and prosthetic work of missing teeth as well as surgical–orthodontic treatment could establish favorable facial esthetics and oral rehabilitation.

## Data Availability

Data sharing does not apply to this article as no datasets were generated or analyzed during the current study.

## Abbreviations

RT: Radiation therapy
PMSO: Posterior maxillary segmental osteotomy
SNA: Sella–nasion–A point angle
SNB: Sella–nasion–B point angle
ANB: A point–nasion–B point angle
Go: Gonion
Me: Menton
U1: Upper central incisor
L1: Lower central incisor
UL: Upper lip
LL: Lower lip
